# Carga de enfermedad tuberculosa atribuible a la diabetes en población adulta de las Américas

**DOI:** 10.26633/RPSP.2017.125

**Published:** 2017-12-19

**Authors:** César Vladimir Munayco, Óscar J. Mújica, Mirtha del Granado, Alberto Barceló

**Affiliations:** 1 Organización Panamericana de la Salud Organización Panamericana de la Salud Washington, DC Estados Unidos de América Organización Panamericana de la Salud, Washington, DC, Estados Unidos de América.

**Keywords:** Tuberculosis, diabetes, comorbilidad, Américas, Tuberculosis, diabetes, comorbidity, Americas, Tuberculose, diabetes, comorbidade, Américas

## Abstract

Se realizó un estudio ecológico para estimar la carga de enfermedad tuberculosa incidente atribuible a la diabetes en la Región de las Américas. El tamaño poblacional, la prevalencia de diabetes y la incidencia de tuberculosis (TB) en la población adulta de cada país de 2013 se emplearon para estimar el riesgo atribuible poblacional porcentual, que ascendió a 16,8% (IC95%: 10,8%- 23,8%), lo que corresponde a 25 045 (16 050-35 741) casos incidentes de TB/año. La diabetes es un importante determinante de la incidencia de tuberculosis en los países de la Región de las Américas y puede dar cuenta de hasta 1 de cada 4 casos incidentes de TB. La intersección de ambas epidemias plantea a los países el doble desafío de la atención y el control integrados de la comorbilidad y de sus determinantes sociales estructurales

## Abstract (en)

An ecological study was conducted to estimate the burden of incident tuberculosis attributable to diabetes in the Region of the Americas.

Population size, the prevalence of diabetes, and the incidence of tuberculosis (TB) in the adult population of each country in 2013 were used to estimate the percent population attributable risk.

The estimated population attributable risk was 16.8% (CI 95%: 10.8-23.8%), which corresponds to 25,045 (16,050-35,741) incident cases of TB per year. Diabetes is an important determinant of the incidence of tuberculosis in the countries of the Region of the Americas and may account for up to 1 in 4 incident TB cases. For countries, the intersection of both epidemics poses the dual challenge of providing integrated are and control of comorbidity and its structural social determinants.

## RESUMO

Estudo ecológico conduzido para estimar a carga de tuberculose incidente atribuível à diabetes na Região das Américas.

O tamanho populacional, a prevalência da diabetes e a incidência de tuberculose na população adulta de cada país em 2013 foram usados para estimar o risco atribuível populacional proporcional.

Estimou-se um risco atribuível populacional de 16,8% (IC95% 10,8%-23,8%), correspondente a 25.045 (16.050-35.741) casos incidentes de tuberculose ao ano.

A diabetes é um importante determinante da incidência de tuberculose nos países da Região das Américas e pode estar associada a até 1 em 4 casos incidentes de tuberculose. A intersecção de ambas as epidemias faz com que os países tenham de enfrentar o duplo desafio da atenção e controle integrados da comorbidade e de seus determinantes sociais estruturais.

La tuberculosis (TB) continúa siendo un problema de salud pública mundial, tanto por su elevada carga de enfermedad como por sus graves consecuencias sociales y económicas, especialmente en países de ingresos medianos y bajos. Pese a los esfuerzos desplegados para reducir la incidencia de TB bajo el sexto Objetivo de Desarrollo del Milenio (ODM), el número estimado de casos incidentes de TB en la Región de las Américas en 2014 aún supera el cuarto de millón (intervalo de confianza de 95%: 270 000-290 000) (1).

A lo largo del tiempo, la epidemia de TB se ha visto afectada por otras epidemias, sobre todo por la pandemia de VIH/SIDA: en los Estados Unidos de América, por ejemplo, el VIH/SIDA junto a otros factores indujo un resurgimiento de la TB entre 1989 y 1999, con un exceso de casos estimados conservadoramente en 67 000 (2). En el África Subsahariana, la epidemia generalizada de VIH/SIDA ha tenido un impacto masivo sobre el crecimiento de los casos de TB, pues se sabe que la infección por VIH incrementa el riesgo de reactivación tuberculosa casi 100 veces en pacientes sin tratamiento antirretroviral (3,4).

En los últimos años, y como consecuencia de notorios cambios transicionales demográficos y epidemiológicos, ha emergido una nueva pandemia: la de diabetes. La globalización de estilos de vida no saludables y de otros determinantes sociales de la salud han ocasionado que la carga mundial de diabetes en la población adulta se incremente de 108 millones de casos en 1980 a 422 millones el 2014 (5). Si las tendencias post-2000 continúan, esta carga mundial sobrepasará los 700 millones en 2025 (6).

Además de las profundas implicaciones de orden sanitario, económico y social que semejante aumento en la carga de enfermedad tiene *per se*, es verosímil considerar que la nueva pandemia de diabetes tenga un impacto marcado sobre la epidemia de TB (7). En un metanálisis se señaló que la diabetes incrementa el riesgo de infección tuberculosa y de tuberculosis activa en aproximadamente tres veces, aunque el rango de las estimaciones del riesgo relativo en los diferentes estudios fue variable (8). A pesar de esta asociación encontrada en estudios epidemiológicos, todavía existen controversias sobre su naturaleza causal: diabetes y tuberculosis; lo que sí es evidente es que los pacientes con TB y diabetes tienen mayor riesgo de fracaso del tratamiento antituberculoso, además de muerte y recaídas (9).

En las Región de las Américas se espera que el impacto de la diabetes en la TB sea mucho mayor que el impacto que sobre ésta tiene la pandemia de VIH, debido a que la prevalencia de diabetes en la población general es mucho mayor que la de VIH. Por otra parte, la mayor carga de diabetes se concentra en zonas urbanas densamente pobladas (10) y afecta a una proporción considerable de personas en situación de pobreza (11), un escenario donde, precisamente, se desarrolla la TB (12).

A lo largo del tiempo, la epidemia de TB se ha visto afectada por otras epidemias, sobre todo por la pandemia de VIH/SIDA: en los Estados Unidos de América, por ejemplo, el VIH/SIDA junto a otros factores indujo un resurgimiento de la TB entre 1989 y 1999, con un exceso de casos estimados conservadoramente en 67 000 (2). En el África Subsahariana, la epidemia generalizada de VIH/SIDA ha tenido un impacto masivo sobre el crecimiento de los casos de TB, pues se sabe que la infección por VIH incrementa el riesgo de reactivación tuberculosa casi 100 veces en pacientes sin tratamiento antirretroviral (3,4).

En los últimos años, y como consecuencia de notorios cambios transicionales demográficos y epidemiológicos, ha emergido una nueva pandemia: la de diabetes. La globalización de estilos de vida no saludables y de otros determinantes sociales de la salud han ocasionado que la carga mundial de diabetes en la población adulta se incremente de 108 millones de casos en 1980 a 422 millones el 2014 (5). Si las tendencias post-2000 continúan, esta carga mundial sobrepasará los 700 millones en 2025 (6).

Además de las profundas implicaciones de orden sanitario, económico y social que semejante aumento en la carga de enfermedad tiene *per se*, es verosímil considerar que la nueva pandemia de diabetes tenga un impacto marcado sobre la epidemia de TB (7). En un metanálisis se señaló que la diabetes incrementa el riesgo de infección tuberculosa y de tuberculosis activa en aproximadamente tres veces, aunque el rango de las estimaciones del riesgo relativo en los diferentes estudios fue variable (8). A pesar de esta asociación encontrada en estudios epidemiológicos, todavía existen controversias sobre su naturaleza causal: diabetes y tuberculosis; lo que sí es evidente es que los pacientes con TB y diabetes tienen mayor riesgo de fracaso del tratamiento antituberculoso, además de muerte y recaídas (9).

En las Región de las Américas se espera que el impacto de la diabetes en la TB sea mucho mayor que el impacto que sobre ésta tiene la pandemia de VIH, debido a que la prevalencia de diabetes en la población general es mucho mayor que la de VIH. Por otra parte, la mayor carga de diabetes se concentra en zonas urbanas densamente pobladas (10) y afecta a una proporción considerable de personas en situación de pobreza (11), un escenario donde, precisamente, se desarrolla la TB (12).

A lo largo del tiempo, la epidemia de TB se ha visto afectada por otras epidemias, sobre todo por la pandemia de VIH/SIDA: en los Estados Unidos de América, por ejemplo, el VIH/SIDA junto a otros factores indujo un resurgimiento de la TB entre 1989 y 1999, con un exceso de casos estimados conservadoramente en 67 000 (2). En el África Subsahariana, la epidemia generalizada de VIH/SIDA ha tenido un impacto masivo sobre el crecimiento de los casos de TB, pues se sabe que la infección por VIH incrementa el riesgo de reactivación tuberculosa casi 100 veces en pacientes sin tratamiento antirretroviral (3,4).

En los últimos años, y como consecuencia de notorios cambios transicionales demográficos y epidemiológicos, ha emergido una nueva pandemia: la de diabetes. La globalización de estilos de vida no saludables y de otros determinantes sociales de la salud han ocasionado que la carga mundial de diabetes en la población adulta se incremente de 108 millones de casos en 1980 a 422 millones el 2014 (5). Si las tendencias post-2000 continúan, esta carga mundial sobrepasará los 700 millones en 2025 (6).

Además de las profundas implicaciones de orden sanitario, económico y social que semejante aumento en la carga de enfermedad tiene *per se*, es verosímil considerar que la nueva pandemia de diabetes tenga un impacto marcado sobre la epidemia de TB (7). En un metanálisis se señaló que la diabetes incrementa el riesgo de infección tuberculosa y de tuberculosis activa en aproximadamente tres veces, aunque el rango de las estimaciones del riesgo relativo en los diferentes estudios fue variable (8). A pesar de esta asociación encontrada en estudios epidemiológicos, todavía existen controversias sobre su naturaleza causal: diabetes y tuberculosis; lo que sí es evidente es que los pacientes con TB y diabetes tienen mayor riesgo de fracaso del tratamiento antituberculoso, además de muerte y recaídas (9).

En las Región de las Américas se espera que el impacto de la diabetes en la TB sea mucho mayor que el impacto que sobre ésta tiene la pandemia de VIH, debido a que la prevalencia de diabetes en la población general es mucho mayor que la de VIH. Por otra parte, la mayor carga de diabetes se concentra en zonas urbanas densamente pobladas (10) y afecta a una proporción considerable de personas en situación de pobreza (11), un escenario donde, precisamente, se desarrolla la TB (12).

El presente estudio tuvo por objetivo cuantificar la carga de enfermedad tuberculosa atribuible a ladiabetes en los países de la Región de las Américas, con el propósito de que esta información contribuya al fortalecimiento de los programas de control de esta emergente comorbilidad.

## MATERIALES Y MÉTODOS

Se diseñó un estudio ecológico de atribución poblacional del riesgo de enfermar de TB condicionado a la prevalencia de diabetes y a la fuerza de la asociación conocida entre ambas enfermedades en la población adulta de 44 países y territorios de la Región de las Américas (que representa 99,9% de la población adulta a nivel regional) correspondiente al año 2013, a partir de fuentes secundarias de datos.

La *población adulta*, definida como aquella comprendida entre los 20 y 79 años de edad, para cada país en 2013 se obtuvo por interpolación lineal anual de las estimaciones de la fecundidad media de la División de Población de Naciones Unidas revisadas a 2012 (13). La *prevalencia de diabetes* (%) en la población adulta (es decir, de 20 a 79 años de edad) para cada país en 2013 se extrajo de las estimaciones nacionales del Atlas de Diabetes de la Federación Internacional de Diabetes (FID) (14). La *incidencia de tuberculosis* (de todas sus formas) por 100 000 habitantes para cada país en 2013 se extrajo del Informe Mundial de Tuberculosis 2014 de la Organización Mundial de la Salud (OMS) (15).

Para cada país/territorio estudiado, se calculó la fracción de casos de TB en la población adulta atribuibles a la diabetes (es decir, la fracción atribuible poblacional) y el número absoluto de casos de TB atribuibles a la diabetes. La fracción atribuible poblacional porcentual (FAP%) se estimó a partir de la fórmula del riesgo atribuible poblacional porcentual modificada por Levin (16-18):

$$FAP\%=\frac{p_{e}\times \left(RR_{e}-1\right)}{p_{e}\times \left(RR_{e}-1\right)+1}\times 100$$

donde *p_e_* representa la prevalencia de diabetes (%) y *RR_e_*, el riesgo relativo de tuberculosis debido a diabetes.

El valor de referencia asumido para el estimador de la fuerza de asociación conocida entre la diabetes y la TB, esto es el *RRe*, fue 3,11 (intervalo de confianza de 95%, IC95%: 2,27-4,26), estimado por Jeon y Murray en 2008 en el único metanálisis publicado hasta la fecha a partir de 13 estudios observacionales sobre esta comorbilidad (8), aunque se han publicado otros estudios posteriormente.

El número absoluto de casos de TB atribuibles a diabetes en cada país/territorio se calculó directamente multiplicando la correspondiente FAP% por el total de casos incidentes de TB en cada país/territorio obtenido por retrocálculo a partir de la tasa de incidencia y del tamaño poblacional respectivos.

Adicionalmente, el análisis por países fue sintetizado para las subregiones geográficas de la Región de las Américas (Norteamérica, Mesoamérica, El Caribe, Sudamérica andina, Sudamérica no andina) Las estimaciones subregionales de prevalencia de diabetes y de incidencia de TB se obtuvieron por suma producto (es decir, el promedio ponderado poblacional) y fueron internamente consistentes con las estimaciones a nivel nacional.

## RESULTADOS

El cuadro 1 presenta las estimaciones puntuales y los IC95% de la fracción atribuible poblacional porcentual y del número absoluto de casos incidentes de tuberculosis atribuibles a la diabetes para cada país, internamente consistentes con sus correspondientes valores de prevalencia de diabetes e incidencia de tuberculosis para 2013. La FAP% fluctuó entre 8,3% (5,2%-12,2%) en Perú hasta 28,2% (19,1%- 37,8%) en Curazao, y el número de casos de TB atribuibles a la diabetes, entre 0 casos en algunas islas del Caribe hasta 9 722 (6 250-13 815) en Brasil. Además de Perú, en los otros tres países con incidencia de tuberculosis mayor de 100 por 100 000 habitantes -Haití, Bolivia y Guyanala FAP% fue 10,5, 11,7 y 22,9%, respectivamente, y el número de casos de TB atribuibles a la diabetes, 1 203, 828 y 107, respectivamente.

En el cuadro 2 se resumen las estimaciones de la FAP% y el número de casos de TB atribuibles a la diabetes agregados a nivel subregional y regional. En la Región de las Américas en conjunto, la prevalencia de diabetes estimada fue 9,6% y la incidencia de TB, 27,6 por 100 000 habitantes. La prevalencia de diabetes más alta se observó en Mesoamérica (10,9%) y Norteamérica (10,8%) y la incidencia de TB más alta, en la subregión del Caribe (62,6 x 100 000) y la Sudamérica andina (61,9 x 100 000). A escala regional, la FAP% estimada de TB atribuible a diabetes fue 16,8% (10,8%-23,8%), cifra que corresponde a 25 045 (16 050-35 741) casos incidentes de tuberculosis en 2013.

## DISCUSIÓN

Este estudio muestra que, en 2013, en la Región de las Américas, aproximadamente 3 de cada 20 casos incidentes de tuberculosis (16,8%) son atribuibles a la presencia de diabetes mellitus en la población adulta. Esta fracción atribuible poblacional de tuberculosis a la diabetes fluctúa desde 1 de cada 10 casos de TB (10,8%) hasta 1 de cada 4 casos de TB (23,8%) dependiendo del nivel de prevalencia de diabetes en la población adulta. En general, se observó que, a mayor prevalencia de diabetes en la población adulta, mayor es el riesgo atribuible poblacional porcentual de TB.

La FAP% estimada en este estudio es similar a la estimada por Lönnroth y colaboradores (19) para la Región de las Américas con datos de 2012 (17%) y comparable en magnitud con la estimada para otras regiones de la OMS (17% en el Mediterráneo Oriental, 16% en el Pacífico Occidental, 15% en Europa, y 14% en el Sudeste Asiático), pero fue mayor que la FAP% estimada para África (9%). Además, la FAP% estimada en el presente estudio es comparable con la FAP% estimada por Lönnroth y colaboradores (19) para China, Rusia, India y Sudáfrica, las FAP% más altas entre los 10 países con incidencia de TB más elevada.

**CUADRO 1.** Fracción atribuible poblacional porcentual (FAP%) y número absoluto de casos de tuberculosis atribuibles a la diabetes por país, las Américas, 2013

País	ISO-3	Población subregional adulta (20-79 años)	Prevalencia subregional de diabetes (%)	Incidencia de tuberculosis (todas las formas por 100 000)	No. de casos nuevos de tuberculosis (20-79 años)	Fracción atribuible poblacional (%)			No. de casos de tuberculosis atribuibles a diabetes mellitus		
						FAP%	Intervalo de confianza de 95%		No. de casos	Intervalo de confianza de 95%	
Anguila	AIA	10 204	13,07	21,0	2	21,6	14,2	29,9	0	0	1
Antigua y Barbuda	ATG	58 080	13,48	13,0	8	22,1	14,6	30,5	2	1	2
Argentina	ARG	26 869 416	5,98	24,0	6 449	11,2	7,1	16,3	723	455	1 052
Aruba	ABW	73 372	17,18	12,0	9	26,6	17,9	35,9	2	2	3
Bahamas	BHS	259 537	14,45	9,8	25	23,4	15,5	32,0	6	4	8
Barbados	BRB	204 415	14,63	1,4	3	23,6	15,7	32,3	1	0	1
Belice	BLZ	182 101	13,42	37,0	67	22,1	14,6	30,4	15	10	21
Bermuda	BMU	48 513	14,86	0	0	23,9	15,9	32,6	0	0	0
Bolivia	BOL	5 742 060	6,29	123,0	7 063	11,7	7,4	17,0	828	522	1 202
Brasil	BRA	131 941 782	9,04	46,0	60 693	16,0	10,3	22,8	9 722	6 250	13 815
Canadá	CAN	25 812 841	10,21	5,0	1 291	17,7	11,5	25,0	229	148	322
Chile	CHL	12 094 034	10,36	16,0	1 935	17,9	11,6	25,2	347	225	489
Colombia	COL	29 978 710	7,12	3,0	9 593	13,1	8,3	18,8	1 253	796	1 807
Costa Rica	CRI	3 226 212	6,78	11,0	355	12,5	7,9	18,1	44	28	64
Cuba	CUB	8 360 691	9,74	9,3	778	17,0	11,0	24,1	133	86	187
Curazao	CUW	112 442	18,65	1,4	2	28,2	19,1	37,8	0	0	1
Dominica	DMA	46 495	11,29	4,8	2	19,2	12,5	26,9	0	0	1
Ecuador	ECU	9 334 995	5,68	56,0	5 228	10,7	6,7	15,6	559	352	817
El Salvador	SLV	3 598 304	9,42	39,0	1 403	16,6	10,7	23,5	233	150	330
Estados Unidos	USA	223 803 222	10,90	3,3	7 386	18,7	12,2	26,2	1 381	898	1 936
Granada	GRD	65 405	8,53	4,1	3	15,3	9,8	21,8	0	0	1
Guatemala	GTM	7 369 065	8,97	60,0	4 421	15,9	10,2	22,6	704	452	1 000
Guyana	GUY	428 141	14,08	109,0	467	22,9	15,2	31,5	107	71	147
Haití	HTI	5 546 066	5,58	206,0	11 425	10,5	6,6	15,4	1 203	756	1 758
Honduras	HND	4 276 211	6,28	54,0	2 309	11,7	7,4	17,0	270	171	392
Islas Caimán	CYM	34 404	14,88	9,8	3	23,9	15,9	32,7	1	1	1
Islas Vírgenes
Británicas	VGB	20 380	12,89	4,1	1	21,4	14,1	29,6	0	0	0
Islas Vírgenes
(EUA)	VIR	74 397	16,10	7,7	6	25,4	17,0	34,4	1	1	2
Jamaica	JAM	1 684 611	10,59	6,5	109	18,3	11,9	25,7	20	13	28
México	MEX	74 127 611	11,77	21,0	15,567	19,9	13,0	27,7	3 097	2 024	4 317
Nicaragua	NIC	3 359 620	10,25	55,0	1 848	17,8	11,5	25,0	329	213	463
Panamá	PAN	2 365 778	7,87	48,0	1 136	14,2	9,1	20,4	162	103	232
Paraguay	PRY	3 834 659	6,17	44,0	1 687	11,5	7,3	16,7	194	123	283
Perú	PER	18 362 172	4,28	124,0	22 769	8,3	5,2	12,2	1 886	1 174	2 788
Puerto Rico	PRI	2 552 317	15,42	1,6	41	24,5	16,4	33,5	10	7	14
República Dominicana	DOM	6 125 678	10,66	60,0	3 675	18,4	11,9	25,8	675	438	948
San Kitts y Nevis	KNA	32 516	13,55	0	0	22,2	14,7	30,6	0	0	0
San Marteen	SXM	25 504	14,79	5,1	1	23,8	15,8	32,5	0	0	0
Santa Lucía	LCA	117 994	8,35	5,7	7	15,0	9,6	21,4	1	1	1
San Vicente y las
Granadinas	VCT	70 051	9,81	24,0	17	17,1	11,1	24,2	3	2	4
Suriname	SUR	337 857	10,87	39,0	132	18,7	12,1	26,2	25	16	34
Trinidad y Tobago	TTO	953 539	13,89	21,0	200	22,7	15,0	31,2	45	30	62
Uruguay	URY	2 264 947	6,34	30,0	679	11,8	7,5	17,1	80	51	116
Venezuela	VEN	18 637 938	6,61	33,0	6 151	12,2	7,7	17,7	753	476	1 090

La FAP% de TB incidente debida a diabetes notificada en estudios realizados en otras latitudes fluctúa según el país estudiado. Por ejemplo, en un estudio realizado en la India (20), la FAP% fue 14,8%, mientras que en otro realizado en Taiwán (21) la diabetes explicó 4% de los casos incidentes de TB. En el presente estudio, todos los países de la Región de las Américas la FAP% fue mayor que la notificada en Taiwán, y solamente en 12 países de los 44 estudiados (Argentina, Bolivia, Colombia, Costa Rica, Ecuador, Haití, Honduras, Panamá, Paraguay, Perú, Uruguay y Venezuela) la FAP% de diabetes para TB fue menor que la notificada para la India.

Es interesante contrastar los hallazgos de este estudio con los del clásico estudio de Lienhardt y colaboradores para estimar el riesgo atribuible poblacional porcentual (RAP%) de TB debida a la infección por VIH/SIDA (22). En su estudio, dicho riesgo fue 4% — considerablemente más bajo que el notificado en la presente investigación para la TB atribuible a la diabetes. Aunque la fuerza de la asociación entre TB e infección por VIH -estimada por el riesgo relativo- es mayor que entre la TB y la diabetes (3,4), probable-mente sea la muy baja prevalencia de infección por VIH en las Región de las Américas -que la clasifica en el escenario de epidemias concentradaslo que explique la baja FAP% de VIH/SIDA para la TB frente a la atribuible a la diabetes en la dinámica de transmisión de TB en la población adulta regional.

**CUADRO 2.** Fracción atribuible poblacional porcentual (FAP%) y número absoluto de casos de tuberculosis atribuibles a la diabetes por subregión, las Américas, 2013

Subregión de las Américas	Población subregional adulta (20–79 años)	Prevalencia subregional de diabetes (%)	Incidencia de tuberculosis (todas las formas por 100 000)	No. de casos nuevos de tuberculosis (20–79 años)	(FAP%)			Casos de tuberculosis atribuibles a diabetes mellitus		
					(FAP%)	Intervalo de confianza de 95%		No. de casos	Intervalo de confianza de 95%	
Norteamérica ^ (1)^	249 664 576	10,83	3,48	8 676	18,6	12,1	26,1	1 610	1 046	2 259
Mesoamérica ^ (2)^	104 448 479	10,91	29,41	30 714	18,7	12,2	26,2	5 513	3 579	7 746
El Caribe ^ (3)^	21 250 519	9,95	62,62	13 307	17,4	11,2	24,5	1 577	1 000	2 278
Sudamérica andina ^ (4)^	82 055 875	6,15	61,91	50 803	11,5	7,2	16,7	5 279	3 320	7 704
Sudamérica no andina ^ (5)^	177 004 838	8,57	40,36	71 444	15,3	9,8	21,8	11 067	7 104	15 755
Región de las Américas	634 424 287	9,58	27,58	174 944	16,8	10,8	23,8	25 045	16 050	35 741

(1) BMU, CAN, USA;

(2) CRI, DOM, GTM, HND, MEX, NIC, PAN, SLV;

(3) AIA, ATG, ABW, BHS, BRB, BLZ, VGB, CYM, CUB, CUW, DMA, GRD, GUY, HTI, JAM, PRI, KNA, LCA, SXM, VCT, SUR, TTO, VIR;

(4) BOL, COL, ECU, PER, VEN;

(5) ARG, BRA, CHL, PRY, URY.

La principal limitación de este estudio -además de su carácter ecológico exploratorioes la inherente restricción de la modelización de la variabilidad de la fuerza de la asociación entre TB y diabetes, que está circunscrita a un único valor puntual (la estimación metanalítica de Jeon y Murray) (8). Ello está relacionado con la falta de estudios regionales disponibles sobre la determinación del riesgo relativo de TB en presencia de diabetes. En consecuencia, no es posible descartar la sobreestimación o subestimación del valor notificado de la FAP% en los países estudiados. La intersección y convergencia de estas dos epidemias contemporáneas plantea un nuevo y doble reto para los sistemas de salud y los programas de control: el de la atención y el control *integrados* de la comorbilidad, incluyendo la acción sobre sus determinantes sociales (23, 24). La estimación del riesgo atribuible poblacional de la diabetes sobre la TB es importante porque permite evaluar el impacto potencial de aquella sobre ésta en un escenario epidemiológico transicional que favorece la sobrecarga de enfermedades crónicas no transmisibles.

Este estudio intenta enriquecer este conocimiento ilustrando la magnitud de la carga de enfermedad tuberculosa evitable por reducción del riesgo de desarrollar diabetes en la población general, suponiendo que esta relación sea causal, además de estimar el número de pacientes con TB y de diabetes que se podrían beneficiar si tuvieran una atención adecuada dual de la diabetes y la tuberculosis, entendida como la mejora de los resultados del tratamiento de la tuberculosis. Finalmente, estudios como el presente pueden facilitar la necesaria abogacía en y con los países y programas orientada a mejorar las estrategias de control de ambas enfermedades, así como de sus determinantes sociales comunes.

### Financiación

Esta publicación ha sido posible gracias al apoyo de la Oficina de Desarrollo Sostenible Regional, Departamento para América Latina y el Caribe, Agencia de los Estados Unidos para el Desarrollo Internacional bajo la Subvención No, AID-LAC-IO-11-00,001.

### Declaración

Las opiniones expresadas por los autores son de su exclusiva responsabilidad y no reflejan necesariamente las opiniones de la Agencia de los Estados Unidos para el Desarrollo Internacional ni los criterios ni la política de la Organización Panamericana de la Salud o de la RPSP/PAJPH.
